# Innovations in the Management of Musculoskeletal Pain With Alpha-Lipoic Acid (IMPALA Trial): Study protocol for a Double-Blind, Randomized, Placebo-Controlled Crossover Trial of Alpha-Lipoic Acid for the Treatment of Fibromyalgia Pain

**DOI:** 10.2196/resprot.7198

**Published:** 2017-03-28

**Authors:** Ian Gilron, Dongsheng Tu, Ronald Holden, Tanveer Towheed, Dan Ziegler, Louie Wang, Roumen Milev, Christopher Gray

**Affiliations:** ^1^ Queen's University Kingston, ON Canada; ^2^ Heinrich Heine University Dusseldorf Germany; ^3^ Kingston General Hospital Kingston, ON Canada

**Keywords:** fibromyalgia, pain, alpha-lipoic acid, antioxidants

## Abstract

**Background:**

Fibromyalgia is a common disorder characterized by chronic widespread pain, sleep disturbance, fatigue, depression, and cognitive dysfunction, resulting in substantial disability. As current analgesics provide incomplete relief and disabling side effects that aggravate fatigue and cognitive dysfunction, there is a need for new pain treatments with better efficacy and tolerability. Alpha-lipoic acid (ALA) is an antioxidant proven effective in painful diabetic neuropathy with minimal side effects.

**Objective:**

We hypothesize that this agent will provide benefits in fibromyalgia because of several similarities with neuropathic pain and also because it does not cause sedation, fatigue, or mental-slowing. To test this, we have designed a clinical trial that will assess pain, side effects, and other outcomes in participants with fibromyalgia.

**Methods:**

Using a crossover design, 24 adults with fibromyalgia will be randomly allocated to 1 of the 2 sequences of ALA and placebo. Participants will take capsules containing ALA or placebo for 4 weeks followed by a 1-week washout followed by a second 4-week treatment and 1-week washout period. ALA (or matching placebo) capsules will be titrated to 1800 mg/day over each 4-week period. The primary outcome will be mean daily pain intensity (0-10) during week 4 of each period. Secondary outcomes, assessed during week 4 of each period, will include global improvement, adverse events, mood, and quality of life.

**Results:**

This trial was registered in the International Standard Randomized Controlled Trial registry March 15, 2016 (Number ISRCTN58259979), and it attained ethics approval on December 3, 2016 (Queen’s University Health Sciences & Affiliated Teaching Hospitals Research Ethics Board protocol number ANAE-287-15). The recruitment started in February 2017.

**Conclusions:**

This trial will provide evidence for the efficacy of ALA in fibromyalgia.

**Trial Registration:**

International Standard Randomized Controlled Trial Number (ISRCTN): 58259979; www.isrctn.com/ISRCTN58259979 (Archived by WebCite at http://www.webcitation.org/6og9JdiyZ)

## Introduction

Fibromyalgia is a multisystem disorder characterized by chronic widespread pain, sleep disturbance, fatigue, irritable bowel syndrome, depressed mood, and cognitive dysfunction, that is reflected in functional disability and impaired quality of life [1,2]. This condition is highly prevalent, with estimates suggesting that 1.6% of men and 4.9% of women are affected [3]; however, the reported prevalence of fibromyalgia is much higher in patients also diagnosed with migraine (22.2%) and low back pain (39%) [4]. Fibromyalgia not only causes suffering, but it is also a substantial financial burden. Canadian estimates put the annual direct health care costs of fibromyalgia at Can $350 million, and annual private insurance costs at Can $200 million [5]. The average 6-month direct and indirect costs of fibromyalgia per person in Canada are Can $2298 and Can $5035, respectively [6]. In the United States, for every dollar spent on fibromyalgia-specific claims, employers spend another US $57-US $143 on additional direct and indirect costs [7]. Thus, in addition to being devastating to the afflicted individual and his or her family, fibromyalgia also exerts a major adverse socioeconomic impact on the society.

Chronic widespread pain is the predominant feature of fibromyalgia [1]. Many drug (eg, nonsteroidal antiinflammatory drugs [NSAIDs], antidepressants, opioids, and anticonvulsants) and nondrug (eg, exercise, acupuncture, cognitive-behavioral therapy) treatments have been evaluated in hundreds of randomized controlled trials (RCTs) [8,9]. In addition to exercise and cognitive behavioral therapy, pharmacotherapy remains an important treatment for fibromyalgia [8]. Evidence-based treatment recommendations from the European League Against Rheumatism, the American Pain Society, and the Association of the Scientific Medical Societies in Germany have included amitriptyline, cyclobenzaprine, tramadol, gabapentin or pregabalin, fluoxetine, and duloxetine [10]. Unfortunately, the current therapies available for treating fibromyalgia do not provide complete relief from all the symptoms. In fibromyalgia (and most other chronic pain conditions), current drugs reduce pain by only 25-40% on average, and meaningful relief occurs in only 40-60% of the patients [9,11]. This is in part due to incomplete efficacy and dose-limiting adverse events associated with these drugs (eg, sedation, cognitive dysfunction, and dizziness). Moreover, some of these side effects (eg, fatigue and cognitive dysfunction) are also common symptoms of the disease, so current drug therapies can actually exacerbate fibromyalgia symptoms. There is some evidence to suggest that combinations of drugs with different mechanisms of action and nonoverlapping side effects may provide superior relief for fibromyalgia patients while minimizing adverse events [12]; however, there are currently very few high-quality trials testing this hypothesis and a related systematic review is currently underway [13].

Careful and extensive clinical observations have identified several similarities between neuropathic pain and fibromyalgia (eg, comorbid depression, disturbed sleep, and similar profiles of sensory symptoms and sensory dysfunction) indicating that considerable similarities and overlap exists between these two conditions, that suggests the possibility of common underlying mechanisms such as central sensitization to nociceptive stimuli [14]. Therefore, it is perhaps not surprising that several treatments effective in neuropathic pain (eg, tricyclic antidepressants, selective norepinephrine reuptake inhibitor [SNRI] antidepressants, gabapentin, and pregabalin) are also effective in fibromyalgia [10]. These converging lines of evidence provide a sound rationale for evaluating new therapies for fibromyalgia that are known to be efficacious in neuropathic pain. One such emerging intervention of interest is antioxidant therapy.

There is growing evidence implicating a role of reactive oxygen species and antioxidants in pain modulation [15-18]. In a mouse pain model, levels of the endogenous antioxidant superoxide dismutase were correlated with the degree of capsaicin-induced hyperalgesia, such that lower antioxidant levels were associated with greater hyperalgesia [19]. These findings suggest that injury-induced pain processing is due in part to accumulation of reactive oxygen species. Furthermore, early preclinical and clinical evidence suggests that various antioxidant compounds have analgesic effects in various pain conditions including vitamin C in complex regional pain syndrome [20], soy protein in neuropathic pain [21], and a combination of different antioxidants in pancreatitis [22]. Alpha-lipoic acid (ALA) has antioxidant activity in its reduced and oxidized forms [23], and is likely the antioxidant that has been studied most extensively for its analgesic efficacy in humans. Over the past several decades, ALA has been studied in the setting of pain in dozens of investigations. Preclinical evidence for analgesic mechanisms of ALA include decreased sensitivity to noxious stimulation through inhibition of T-type calcium (Cav3.2) channels [24]. Clinically, at least sixteen RCTs of ALA involving more than 1320 participants have reported reductions in pain and other diabetic neuropathy symptoms [25-28]. Although symptomatic improvement with ALA has been demonstrated mostly in diabetic neuropathy populations, evidence also suggests the potential for efficacy in other pain conditions such as chemotherapy-induced neuropathy [29,30] and burning mouth syndrome [31].

Current fibromyalgia treatments provide clinically relevant pain relief in only some patients and also frequently exacerbate other disabling features of fibromyalgia. An analgesic agent that does not cause central nervous system depression would be of great benefit to patients suffering from fibromyalgia. Unfortunately, there is no evidence to support the efficacy of acetaminophen or NSAIDs in fibromyalgia, and any potential benefits of NSAIDs are greatly outweighed by their gastrointestinal and other adverse effects such that NSAIDs are recommended against in recent fibromyalgia guidelines [10]. Thus, there is clearly a desperate need for new fibromyalgia treatments with greater analgesic efficacy, but also with a better safety and tolerability profile that is best suited to the constellation of fibromyalgia-related symptoms. ALA has demonstrated safety and efficacy (number-needed-to-treat=~6) in neuropathic pain in 16 RCTs of more than 1320 patients to date [27]. Given the similarities between neuropathic pain conditions and fibromyalgia, we hypothesize that ALA will be effective in reducing pain in fibromyalgia patients. Furthermore, given that ALA is nonsedating with no reported fatigue or cognitive dysfunction, we expect that ALA will have the added benefit of not exacerbating these fibromyalgia-related symptoms.

## Methods

### Ethics Approval

This study underwent ethics review and received a compliance notice from the Queen’s University Health Sciences & Affiliated Teaching Hospitals Research Ethics Board. This study protocol will be conducted in accordance with the principles of the Declaration of Helsinki and also consistent with the International Council for Harmonisation Good Clinical Practice: Consolidated guideline.

### Aims and Hypothesis

The objective of this trial was to evaluate the safety and efficacy of ALA in treating pain in participants with fibromyalgia. Our primary hypothesis is that ALA is safe and superior to placebo in treating pain in fibromyalgia.

### Design

**Figure 1:**
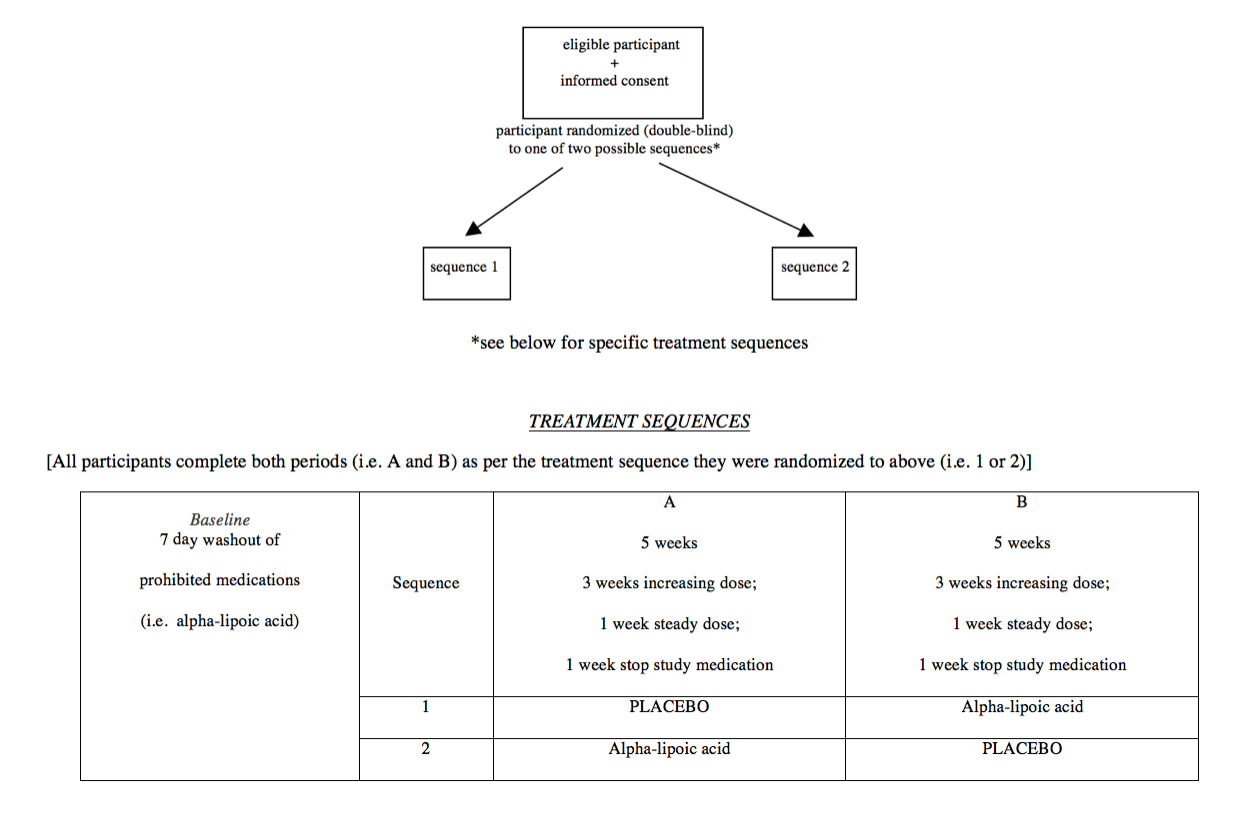
Flow of participants through the trial.

This is a double-blind, randomized, 2-period crossover controlled trial comparing ALA to placebo in adults with fibromyalgia (Figure 1). Each of the 2 treatment periods will be 5 weeks in duration, and the entire trial will be 10 weeks long for each participant. Participants will be randomized to one of the two treatment sequences. The randomization sequence will be generated using the Web-based program—randomization.com (Dallal, Tufts University). Participants in sequence 1 will take active inert placebo capsules during the first 4 weeks of the trial (followed by a 1-week washout period) and will subsequently take active ALA capsules for the next 4 weeks of the trial (followed by a 1-week washout period). Participants in sequence 2 will take active ALA capsules during the first 4 weeks of the trial (followed by a 1-week washout period) and will subsequently take inert placebo capsules for the next 4 weeks of the trial (followed by a 1-week washout period). See Figure 1 for a trial design schematic.

**Figure 2:**
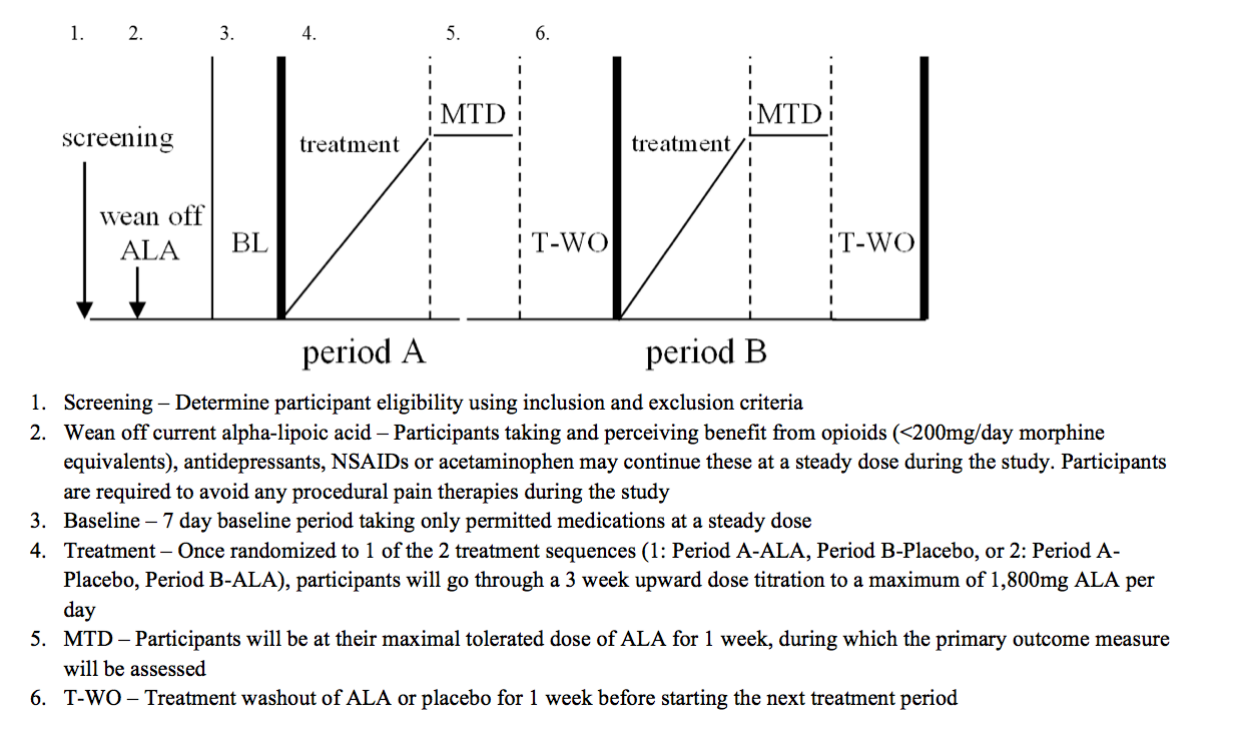
Trial design schematic representation.

### Setting

Investigators work in a tertiary care health sciences center in Kingston, Ontario, Canada.

### Participants

Men and women aged 18 years and older meeting the 2016 American College of Rheumatology revised diagnostic criteria for fibromyalgia will be considered for recruitment following informed consent. The inclusion and exclusion criteria are given in Textboxes 1 and .

**Inclusion criteria for the study.**

- Adults aged 18 years and older
- Diagnosed with fibromyalgia using the 2016 American College of Rheumatology revised diagnostic criteria
- Experiencing daily moderate pain (≥4/10 on a Numerical Rating Scale) for at least three months
- Women of childbearing potential must have a negative serum beta-human chorionic gonadotropin (HCG) test and are required to use a highly effective form of contraception while on trial
- Have the necessary abilities, visual acuity, and English language skills to complete questionnaires and pain diaries and to participate in telephone communication with study nurses to permit titration of the study drugs

**Exclusion criteria for the study.**

- Presence of a painful condition, including inflammatory rheumatic disease, more than 50% as severe as, but distinct from, fibromyalgia
- Women who are pregnant or lactating
- Women of childbearing potential not using adequate contraceptives
- End-stage kidney or liver disease
- Unstable cardiovascular disease (myocardial infarction within the preceding year, unstable angina, or congestive heart failure) or clinically relevant abnormal 12-lead electrocardiogram
- Any poorly controlled medical condition that, in the opinion of the investigator, would interfere with proper conduct of the trial
- Severe depression, as determined by a Beck Depression Inventory-II score of 29 or more; suicidal ideation, as determined by a Beck Depression Inventory score of 2 or more; any current major psychiatric disorder (eg, schizophrenia, bipolar disorder, and so on) that is not well-controlled
- Hypersensitivity to any of the study medications
- Any current alcohol or drug abuse or dependence (except nicotine and caffeine). Participants with a history of abuse/dependence with more than 1 year of abstinence may be considered for inclusion
- Those taking more than 90 mg morphine equivalents per day

### Randomization and Blinding

We will use a balanced Latin Square crossover design in which participants will be allocated to one of the two treatment sequences of ALA and placebo. At the beginning of the trial, an investigational pharmacist will use a computer-generated randomization process to prepare a concealed allocation schedule to randomly assign the treatment sequences, in appropriate block sizes, to a consecutive series of numbers. On enrollment, each participant will be assigned to the next consecutive number, and the corresponding series of study medications will be dispensed (eg, ALA followed by placebo or placebo followed by ALA). Study medications will be formulated in an identical fashion across treatment periods. Treatment codes for each study participant will be generated by an investigational pharmacist and will not be disclosed to study personnel or participants until completion of all participants in the trial. Study outcome measures will be evaluated and recorded by the research study nurse who will be blinded (as will the rest of the research team) to treatment group assignments until trial completion. As an assessment of blinding to treatment group, each participant and the study nurse will complete a blinding questionnaire at the end of each treatment period.

### Cointerventions and Rescue Medication

Any enrolled participants already taking ALA will be weaned off during a pretrial washout of at least seven days. Participants taking and perceiving benefit from opioids (<90 mg morphine equivalents), antidepressants (tricyclic, selective serotonin reuptake inhibitor, or SNRI), NSAIDS, or acetaminophen may continue these medications at a steady daily dose for the duration of the study. Any ongoing cognitive-behavioral therapy and exercise programs perceived as beneficial will be allowed to continue only if it is certain that these will be evenly used throughout the trial. Participants will not be allowed to start new medications, cognitive-behavioral therapy, or exercise programs at any point during the study. Participants will be required to avoid any procedural pain therapies (eg, neurosurgical interventions, nerve blocks, or acupuncture) during the study. Participants will be permitted to take acetaminophen (≤8 tablets, 325 mg/tablet daily) for inadequate pain relief only during the taper and washout phases of each treatment period. Acetaminophen consumption will be recorded as a secondary outcome measure.

### Study Treatment Dosing Schedule

ALA active study medication and matching placebo is being supplied by SiSU Incorporated (Burnaby BC, Canada) and encapsulated by Central Medical Pharmacy Inc (Toronto, ON, Canada). During each period of this trial, participants will receive 1 set of capsules (ALA capsules) containing ALA 300 mg or placebo (lactose capsules) to be taken 30 min before meals. Each period will last 5 weeks, with a 4-week treatment period and a 1-week washout period. During week 1 of each treatment period, participants will take 2 capsules before bedtime. During week 2, participants will take 2 capsules at dinnertime and 2 capsules before bedtime. During weeks 3 and 4, participants will take 2 capsules in the morning, 2 capsules at dinnertime, and 2 capsules before bedtime. On the basis of previous studies, we expect virtually all trial participants to tolerate and reach the ceiling dose of ALA 1800 mg/day. However, during this flexible dose titration, the final dose arrived at during the maximal tolerated dose week (week 4 of the treatment period) could be lower than the ceiling dose of 1800 mg, if side effects encountered during the dose titration (eg, nausea) are suspected to be due to ALA. Thus, this trial will not use a forced titration to the ceiling dose of 1800 mg/day. Week 5 would be a complete washout, and participants will take no study medication.

### Outcome Measures

During the trial, the study nurse will contact participants by telephone at least once a week to evaluate adverse effects, assess pain intensity, and encourage compliance. Furthermore, participants will be evaluated in the clinic on 1 of the 5 weekdays of week 4 of each treatment period for vital signs and assessment of secondary outcomes. Finally, participants will be followed up by telephone 2 weeks and 3 months following the completion of the study to document any subsequent problems or adverse events. See Table 1 for the schedule of study assessments.

**Table 1:** Schedule of assessments.

Assessment	Screen	Baseline	Treatment periods			3-Month Posttrial completion
			Weeks 1-3	Week 4	Week 5 (Washout)	
Days per treatment period	−14	−7	1-21	22-28	29-35	
Present pain intensity, 0-10 numerical rating scale (average and worst)	*^b^					
Concurrent medications^a^	*	*	*	*	*	*
Demographics and medical history	*					
Vital signs and weight	*			*		
Clinical biochemistry	*					
Adverse events^a^	*	*	*	*	*	*
Other adverse effects^a^			*	*	*	
Drug dispensing		*		*		
Drug compliance and accountability				*		
Daily pain diaries		*	*	*	*	
Maximal tolerated dose levels			*	*	*	
Medical Outcomes Study-Sleep Scale		*		*		
Patient global impression of change			*	*	*	
Brief Pain Inventory		*		*		
Beck Depression Inventory-II	*	*		*		
Medical Outcomes Study 36-item short-form health survey		*		*		
Rescue acetaminophen^a^			*	*	*	
Fibromyalgia Impact Questionnaire		*		*		
Short-form McGill Pain Questionnaire		*		*		
Blinding questionnaire				*		

^a^Evaluated during weekly participant telephone contacts with research nurse.

^b^* indicates timing of each trial assessment.

The primary outcome is the mean daily “average” pain intensity experienced while on the maximal tolerated dose of ALA or placebo during week 4 (days 22-28). This will be determined from participants’ ratings of their “average pain over the last 24 hours” completed in patient diaries every morning using a numerical rating scale from 0 to 10. Secondary outcomes include: frequency or severity of treatment-emergent adverse effects, Fibromyalgia Impact Questionnaire, Medical Outcomes Study Sleep Scale, Patient Global Impression of Change, Brief Pain Inventory, Beck Depression Inventory-II, Beck Anxiety Inventory, the short form McGill Pain Questionnaire, the SF-36 survey, blinding questionnaires, and acetaminophen consumption. All these outcomes will be assessed at the baseline and during week 4 of each treatment period, except for adverse effects and acetaminophen consumption, which will be assessed weekly during each treatment period. In addition, plasma RNA samples will be collected to search for transcription and protein markers of treatment response.

### Sample Size

Statistical considerations underlying this sample size calculation are based on the null hypothesis that there is no difference in pain intensity between the study treatments and the alternative hypothesis that ALA is different from placebo. Systematic reviews of placebo-controlled chronic pain trials consistently reveal that statistically significant treatment-placebo group differences vary between 0.5 and 1.5 points depending on the magnitude of placebo response in any given trial [32]. Thus, on the basis of previous estimates of within-participant variation in fibromyalgia [33], we project that a sample size of 21 participants would provide an 80% chance of detecting (at an alpha level of .05) a mean treatment group difference of 1.5 points on a 0-10 numerical rating scale. In order to have a sample size divisible by 4, we have adjusted the sample size to 24 participants. Accounting for trial dropout rates from our previous trials and for a 2-period crossover design, we expect that the recruitment of 30 enrollees for each trial will yield the above number of completers.

### Recruitment

One-month lead time will be allowed to begin recruiting study participants. A maximum of 15 participants will be on each treatment period at any given time. On the basis of these factors, our projected trial completion time is 24 months. As we have been doing for our previous and ongoing chronic pain trials [34-37], a concurrent series of participant recruitment methods will be used [38].

### Statistical Analysis

Participants who complete both treatment periods will be included in trial efficacy analyses. When data from only one period are available, sensitivity analysis including all participants will also be performed, by assuming some reasonable but extreme values for the remaining periods. Those receiving at least one dose of study drug will be included in the safety analyses.

The primary outcome will be calculated as an average of pain scores as recorded in the participant pain diaries within the last 7 days (at maximal tolerated dose in week 4 of the treatment), if more than 50% of the information (at least four days) is not missing [39]. Otherwise, mean daily pain will be treated as missing data. Sensitivity analyses on the basis of the average of all available pain scores will also be performed to confirm the results of the primary analysis. A linear mixed model with sequence, period, treatment, and the first order carryover as fixed effects and participant as a random effect [39] will be used to test whether there is any treatment difference among groups and to estimate the least square mean of the mean daily pain intensity for each treatment group, adjusting the carryover and period effects. The pairwise comparison between ALA and placebo will be performed on the basis of the least square means and standard deviations from the linear mixed model. Sensitivity analyses will be performed using a pattern-mixture model [40] on the basis of patterns of missing data so as to check the robustness of results in the case that data may not be missing at random. A Fisher’s Least Significant Difference [41] procedure will be used to adjust the *P* values for ALA versus placebo comparisons.

Secondary outcomes will be analyzed similarly except that (1) only 1 measurement will be analyzed in the last week for the singular measures (ie, final week questionnaires), and (2) the scoring algorithms developed for the Brief Pain Inventory, the Beck Depression Inventory-II, the Beck Anxiety Inventory, and the SF-36 will be first used to derive the subscales or domains within these instruments and the scores on these subscales or domains will be used as response variables in the linear mixed model analysis.

## Results

This trial has been funded by the Physicians’ Services Incorporated Foundation. It was registered in the International Standard Randomized Controlled Trial registry March 15, 2016 (Number ISRCTN58259979), and it attained ethics approval on December 3, 2016 (Queen’s University Health Sciences & Affiliated Teaching Hospitals Research Ethics Board protocol number ANAE-287-15). The recruitment started in February 2017.

## Discussion

### Trial implications

Fibromyalgia remains a challenging condition to treat, with current recommended pharmacological therapies providing only partial relief from pain, and sometimes exacerbating other symptoms. To the best of our knowledge, this proposed trial is the first to investigate the safety and efficacy of the antioxidant, ALA, for the treatment of fibromyalgia pain. As ALA has shown promise in patients with neuropathic pain, which has similar features to fibromyalgia, we expect this antioxidant to provide pain relief with minimal side effects in patients suffering from fibromyalgia.

As with all clinical trials, possible threats to our proposed study include problems with patient recruitment, noncompliance, protocol violations, and early dropouts. However, we are confident that our study design and our experience in leading chronic pain RCTs in this region will mitigate these threats. Furthermore, as in our previous trials, noncompliance, protocol violations, and early dropouts will be minimized by our proposed crossover design, thorough study participant teaching, and close weekly follow up of participants.

Given the current lack of, and desperate need for, new improved fibromyalgia treatments, this research is expected to provide rigorous evidence for a safer and more effective treatment strategy for fibromyalgia. The development of this proof-of-concept RCT of ALA in fibromyalgia will facilitate future confirmatory RCTs and the implementation of ALA into clinical practice such that its benefits may be realized by patients globally.

### Consent for Publication

We will obtain informed consent from all trial participants.

## Abbreviations

ALA: alpha-lipoic acid
NSAID: nonsteroidal antiinflammatory drug
SNRI: selective norepinephrine reuptake inhibitor

## References

[1] Wolfe F (2009). Fibromyalgia wars. J Rheumatol.

[2] Clauw D (2014). Fibromyalgia: a clinical review. J Am Med Assoc.

[3] Gran JT (2003). The epidemiology of chronic generalized musculoskeletal pain. Best Pract Res Clin Rheumatol.

[4] Ifergane G, Buskila D, Simiseshvely N, Zeev K, Cohen H (2006). Prevalence of fibromyalgia syndrome in migraine patients. Cephalalgia.

[5] White KP, Speechley M, Harth M, Ostbye T (1999). The London Fibromyalgia Epidemiology study: direct health care costs of fibromyalgia syndrome in London, Canada. J Rheumatol.

[6] Penrod JR, Bernatsky S, Adam V, Baron M, Dayan N, Dobkin PL (2004). Health services costs and their determinants in women with fibromyalgia. J Rheumatol.

[7] Silverman S, Dukes EM, Johnston SS, Brandenburg NA, Sadosky A, Huse DM (2009). The economic burden of fibromyalgia: comparative analysis with rheumatoid arthritis. Curr Med Res Opin.

[8] Goldenberg DL, Burckhardt C, Crofford L (2004). Management of fibromyalgia syndrome. J Am Med Assoc.

[9] Lunn MP, Hughes RA, Wiffen PJ (2009). Duloxetine for treating painful neuropathy or chronic pain. Cochrane Database Syst Rev.

[10] Häuser W, Thieme K, Turk DC (2010). Guidelines on the management of fibromyalgia syndrome - a systematic review. Eur J Pain.

[11] Häuser W, Bernardy K, Uçeyler N, Sommer C (2009). Treatment of fibromyalgia syndrome with gabapentin and pregabalin--a meta-analysis of randomized controlled trials. Pain.

[12] Gilron I, Chaparro LE, Tu D, Holden RR, Milev R, Towheed T, DuMerton-Shore D, Walker S (2016). Combination of pregabalin with duloxetine for fibromyalgia: a randomized controlled trial. Pain.

[13] Gilron I, Shum B, Moore R, Wiffen P (2013). Combination pharmacotherapy for the treatment of fibromyalgia (Protocol). Cochrane Database Syst Rev.

[14] Koroschetz J, Rehm SE, Gockel U, Brosz M, Freynhagen R, Tölle TR, Baron R (2011). Fibromyalgia and neuropathic pain--differences and similarities. A comparison of 3057 patients with diabetic painful neuropathy and fibromyalgia. BMC Neurol.

[15] Gao X, Kim HK, Chung JM, Chung K (2007). Reactive oxygen species (ROS) are involved in enhancement of NMDA-receptor phosphorylation in animal models of pain. Pain.

[16] Kim HK, Kim JH, Gao X, Zhou J, Lee I, Chung K, Chung JM (2006). Analgesic effect of vitamin E is mediated by reducing central sensitization in neuropathic pain. Pain.

[17] Salvemini D, Little JW, Doyle T, Neumann WL (2011). Roles of reactive oxygen and nitrogen species in pain. Free Radic Biol Med.

[18] Wang Z, Porreca F, Cuzzocrea S, Galen K, Lightfoot R, Masini E, Muscoli C, Mollace V, Ndengele M, Ischiropoulos H, Salvemini D (2004). A newly identified role for superoxide in inflammatory pain. J Pharmacol Exp Ther.

[19] Schwartz ES, Kim HY, Wang J, Lee I, Klann E, Chung JM, Chung K (2009). Persistent pain is dependent on spinal mitochondrial antioxidant levels. J Neurosci.

[20] Zollinger PE, Tuinebreijer WE, Kreis RW, Breederveld RS (2009). Effect of vitamin C on frequency of reflex sympathetic dystrophy in wrist fractures: a randomized trial. Lancet.

[21] Shir Y, Raja SN, Weissman CS, Campbell JN, Seltzer Z (2001). Consumption of soy diet before nerve injury preempts the development of neuropathic pain in rats. Anesthesiology.

[22] Kirk GR, White JS, McKie L, Stevenson M, Young I, Clements WD, Rowlands BJ (2006). Combined antioxidant therapy reduces pain and improves quality of life in chronic pancreatitis. J Gastrointest Surg.

[23] Packer L, Witt EH, Tritschler HJ (1995). alpha-Lipoic acid as a biological antioxidant. Free Radic Biol Med.

[24] Lee WY, Orestes P, Latham J, Naik AK, Nelson MT, Vitko I, Perez-Reyes E, Jevtovic-Todorovic V, Todorovic SM (2009). Molecular mechanisms of lipoic acid modulation of T-type calcium channels in pain pathway. J Neurosci.

[25] Han T, Bai J, Liu W, Hu Y (2012). A systematic review and meta-analysis of α-lipoic acid in the treatment of diabetic peripheral neuropathy. Eur J Endocrinol.

[26] Mijnhout GS, Kollen BJ, Alkhalaf A, Kleefstra N, Bilo HJ (2012). Alpha lipoic Acid for symptomatic peripheral neuropathy in patients with diabetes: a meta-analysis of randomized controlled trials. Int J Endocrinol.

[27] Ziegler D, Nowak H, Kempler P, Vargha P, Low PA (2004). Treatment of symptomatic diabetic polyneuropathy with the antioxidant alpha-lipoic acid: a meta-analysis. Diabet Med.

[28] Ziegler D, Low PA, Litchy WJ, Boulton AJ, Vinik AI, Freeman R, Samigullin R, Tritschler H, Munzel U, Maus J, Schütte K, Dyck PJ (2011). Efficacy and safety of antioxidant treatment with α-lipoic acid over 4 years in diabetic polyneuropathy: the NATHAN 1 trial. Diabetes Care.

[29] Joseph EK, Chen X, Bogen O, Levine JD (2008). Oxaliplatin acts on IB4-positive nociceptors to induce an oxidative stress-dependent acute painful peripheral neuropathy. J Pain.

[30] Guo Y, Jones D, Palmer J, Forman A, Dakhil S, Velasco M, Weiss M, Gilman P, Mills GM, Noga SJ, Eng C, Overman MJ, Fisch MJ (2014). Oral alpha-lipoic acid to prevent chemotherapy-induced peripheral neuropathy: a randomized, double-blind, placebo-controlled trial. Support Care Cancer.

[31] Zakrzewska J, Forssell H, Glenny A (2005). Interventions for the treatment of burning mouth syndrome. Cochrane Database Syst Rev.

[32] Dworkin RH, Turk DC, McDermott MP, Peirce-Sandner S, Burke LB, Cowan P, Farrar JT, Hertz S, Raja SN, Rappaport BA, Rauschkolb C, Sampaio C (2009). Interpreting the clinical importance of group differences in chronic pain clinical trials: IMMPACT recommendations. Pain.

[33] Russell IJ, Mease PJ, Smith TR, Kajdasz DK, Wohlreich MM, Detke MJ, Walker DJ, Chappell AS, Arnold LM (2008). Efficacy and safety of duloxetine for treatment of fibromyalgia in patients with or without major depressive disorder: results from a 6-month, randomized, double-blind, placebo-controlled, fixed-dose trial. Pain.

[34] Gilron I, Bailey JM, Tu D, Holden RR, Weaver DF, Houlden RL (2005). Morphine, gabapentin, or their combination for neuropathic pain. N Engl J Med.

[35] Gilron I, Bailey J, Tu D, Holden R, Jackson A, Houlden R (2009). Nortriptyline and gabapentin, alone and in combination for neuropathic pain: a double-blind, randomised controlled crossover trial. Lancet.

[36] Gilron I, Tu D, Holden RR, Jackson AC, DuMerton-Shore D (2015). Combination of morphine with nortriptyline for neuropathic pain. Pain.

[37] Gilron I, Chaparro L, Tu D, Holden R, Milev R, Towheed T, DuMerton-Shore D, Walker S (2016). Combination of pregabalin with duloxetine for fibromyalgia: a randomized controlled trial. Pain.

[38] Spilker B, Cramer J (1992). Patient recruitment in clinical trials.

[39] Fairclough D, Fayers PM, Hays RD (2005). Analyzing Studies with Missing Data. Assessing Quality of Life in Clinical Trials. 2nd Ed.

[40] Molenberghs G, Kenward M (2007). Missing Data in Clinical Studies Vol. 61.

[41] Hochberg Y, Tamhane A (1987). Multiple comparison procedures.

